# Does Bipedality Predict the Group-Level Manual Laterality in Mammals?

**DOI:** 10.1371/journal.pone.0051583

**Published:** 2012-12-12

**Authors:** Andrey Giljov, Karina Karenina, Yegor Malashichev

**Affiliations:** 1 Department of Vertebrate Zoology, Saint-Petersburg State University, St. Petersburg, Russia; 2 Department of Embryology, Saint-Petersburg State University, St. Petersburg, Russia; University of Lethbridge, Canada

## Abstract

**Background:**

Factors determining patterns of laterality manifestation in mammals remain unclear. In primates, the upright posture favours the expression of manual laterality across species, but may have little influence within a species. Whether the bipedalism acts the same in non-primate mammals is unknown. Our recent findings in bipedal and quadrupedal marsupials suggested that differences in laterality pattern, as well as emergence of manual specialization in evolution might depend on species-specific body posture. Here, we evaluated the hypothesis that the **postural characteristics** are the key variable shaping the manual laterality expression across mammalian species.

**Methodology/Principal Findings:**

We studied forelimb preferences in a most bipedal marsupial, brush-tailed bettong, *Bettongia penicillata* in four different types of unimanual behavior. The significant left-forelimb preference at the group level was found in all behaviours studied. In unimanual feeding on non-living food, catching live prey and nest-material collecting, all or most subjects were lateralized, and among lateralized bettongs a significant majority displayed left-forelimb bias. Only in unimanual supporting of the body in the tripedal stance the distribution of lateralized and non-lateralized individuals did not differ from chance. Individual preferences were consistent across all types of behaviour. The direction or the strength of forelimb preferences were not affected by the animals’ sex.

**Conclusions/Significance:**

**O**ur findings support the hypothesis that the expression of manual laterality depends on the species-typical postural habit. The interspecies comparison illustrates that in marsupials the increase of bipedality corresponds with the increase of the degree of group-level forelimb preference in a species. Thus, bipedalism can predict pronounced manual laterality at both intra- and interspecific levels in mammals. We also conclude that quadrupedal position in biped species can slightly hinder the expression of manual laterality, but the evoked biped position in quadrupedal species does not necessarily lead to the enhanced manifestation of manual laterality.

## Introduction

Asymmetry in motor activity, especially in the use of the limbs, appeared to be much more widespread among vertebrates than previously thought [1]–[3]. This fact is clearly illustrated by the numerous reports showing limb preferences in different taxonomic groups of vertebrate animals ranging from fish [4], [5], amphibians (e.g., [6], [7]), and reptiles [8], [9] to birds (reviewed in [10]–[13]) and mammals (primates: reviewed in [14]–[16], non-primate taxa: e.g., [17]–[21]). The best known example of laterality in forelimb use is a species-typical right-handedness, which was claimed to be characteristic of humans in different historical eras and geographical regions ([22–24; but see [25] for cultural variations). It has been suggested that the emergence of human handedness is related to acquisition of bipedalism [26], [27]. The growing body of evidence showing the postural effect on manual laterality in non-human primates supports this hypothesis.

The upright posture of a subject was found to be correlated to the increased preference in one hand use in many species of prosimians (e.g., [28]–[30]), monkeys (e.g., [26], [31]–[34] and apes (e.g., [35]–[38]). The postural effect was showed not only at the within-subject level, but also when comparing the primate species, which differ in their relative body orientation and postural habit. In prosimian primates the strength of motor laterality increases in a row of six species from the strongly quadrupedal mouse lemurs, *Microcebus murinus*, with a horizontal orientation of the body’s long axis, to the more bipedal galagos, *Galago senegalensis* and *G. moholi*, which typically rest or feed vertically and move by vertical clinging and leaping [29], [30], [39]. This result allowed authors to propose that the species-typical postural orientation contributes to the strength of lateral bias in a given species; in particular, the vertical posture and bipedality favours the manifestation of laterality. Moreover, primate quadrupeds with the horizontal long body axis, such as mouse lemurs, exhibit no increase of manual preferences even when shifting from quadrupedal to vertical or bipedal positions [40], that is, the species-typical posture may have more influence on the laterality and, therefore, has stronger predictive power than within-subject effect of postural change.

The tendency of more bipedal species to be more lateralized in forelimb use could also be traced among apes. The species characterized by relatively higher degree of bipedality, such as chimpanzees, *Pan troglodytes*, and bonobos, *Pan paniscus* [41], tend to exhibit more pronounced manual preferences in most tasks explored than more quadrupedal gorillas, *Gorilla gorilla*, and orang-utans, *Pongo pygmaeus* [15], [36], [42], [43]. Meanwhile, gorillas and gibbons, *Hylobates lar*, are more frequently observed bipedally than orangutans, and at the same time are more liable to express population-level lateral bias in hand use [35].

The mechanism through which animal’s posture affects manual preferences is not clear. It has been suggested that the extreme postural adjustment during manipulation in unstable bipedal stance leads to a general systemic arousal and the increased integration of the nervous system for balance control. These processes can potentially be reflected in the enhanced manifestation of lateralized motoric behaviours [28], [30]. In addition, the effect of posture on manual laterality in monkeys and apes has been suggested to be associated with the changes in grip types preferred by subjects when reaching from a bipedal compared to a quadrupedal posture [36], [44]. It was found that the grip morphology influences the direction of hand preferences in primates [45], [46] and the type of the grip used to grasp a food item can be different as a function of posture [36], [44].

Until recently, the influence of body posture on asymmetry of motor actions in non-primate mammals has not been assessed. It was showed that in obligatory quadrupedal domestic cats, *Felis silvestris catus*, task’s postural demands did not affect either direction or strength of paw preferences [47]. The reaching of food from unstable body posture (vertical clinging) was revealed to be significantly more difficult for experimental subjects as compared to the stable one (sitting or standing); however, no population-level bias was found in either task. Most recently, the similar picture was observed in the quadrupedal tree shrews, *Tupaia belangeri*. Here, also no influence of subjects’ body posture on forelimb preferences was found and lateral bias was expressed at the individual but not at the population level [48]. It was further suggested that the absence of influence of the postural demand on the strength of manual laterality is typical for quadruped non-primate mammals with a horizontal body orientation [48].

The other non-primate species studied in the aspect of within-subject postural effect on laterality was a marsupial mammal, the red-necked wallaby, *Macropus rufogriseus*. Here, much like in primates, the bipedal posture was found to favour the expression of laterality in unimanual behaviours [49]. For instance, when feeding bipedally red-necked wallabies displayed pronounced population-level forelimb preference with the majority of individuals (81%) being lateralized. In feeding from the initial quadrupedal position, to the contrary, no population lateral bias was revealed and only 11% of individuals exhibited significant forelimb preferences. The postural effect at the between-species level in marsupials has also been traced [21]. It was shown that the proportion of lateralized individuals and the strength of forelimb preferences tend to be enhanced with more vertical and bipedal species-typical posture. In addition, in all three species of marsupial quadrupeds studied to date no significant population bias in forelimb use was found [21], [50], whereas the primary bipedal red-necked wallabies did show forelimb preference at the population level. Thus, it was hypothesized that species postural characteristics serve as a factor shaping the manual laterality manifestation in marsupials [21]. However, a small number of marsupial species studied, especially those with bipedal locomotion, limits reliable evolutionary interpretations, and further investigation are clearly required to test this hypothesis.

Therefore, we aimed to explore forelimb preferences in a highly bipedal marsupial, brush-tailed bettong (“woylie”), *Bettongia penicillata*, in four different types of unimanual actions: feeding on non-living food, catching live insects, nest-material collecting, and supporting of the body in the tripedal stance. Such actions were not artificially evoked, but were video recorded during animals’ usual activity in zoos. The possible effects of task and subjects’ sex were tested.

The chosen species, *B. penicillata*, is a member of the Potoroidae (Diprotodontia), containing potoroos and bettongs, also known as rat-kangaroos. Brush-tailed bettongs use bipedal locomotion as their primary gait and move on all four legs only at very slow speeds [51], [52]. According to our hypothesis of the species-typical posture effect on laterality in marsupials [21] the prediction can be done that, being characterized by a high degree of bipedality, brush-tailed bettongs should exhibit pronounced forelimb preferences at both the individual and the group levels.

## Results

### 1. Distribution of Individual Preferences

**Figure 1:**
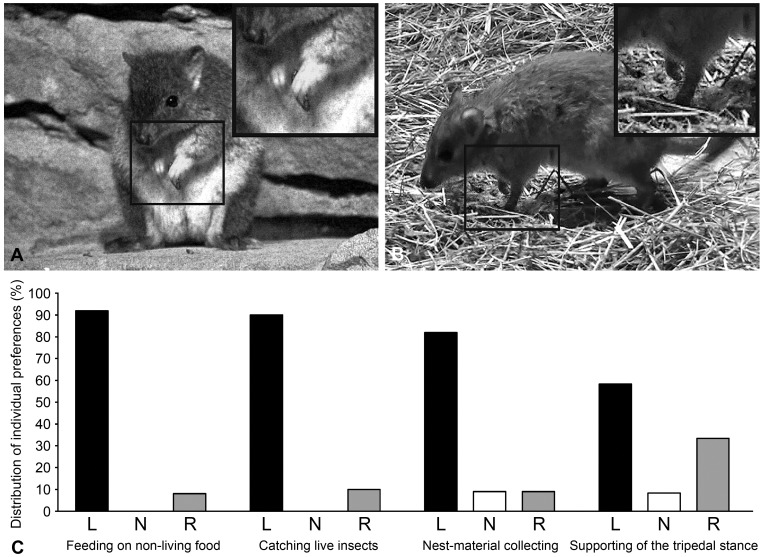
Unimanual forelimb use in brush-tailed bettongs. Two examples of behaviours investigated: (*a*) feeding on non-living food, (*b*) supporting the body in the tripedal stance. Framed body areas are shown enlarged at the corner insertions. (*c*) Percentage distribution of left-forelimb preferent (L), right-forelimb preferent (R) and non-preferent (N) individuals for unimanual feeding on non-living food (*n* = 12), catching live insects (*n* = 10), nest-material collecting (*n* = 11) and supporting of the tripedal stance (*n* = 12).

The individual HI values and z-scores in feeding on non-living food are shown in Table 1. A total of 31 unimanual act per individual was obtained after reduction to the smallest value in the group (see Methods) for feeding on non-living food. According to individual *z* scores all 12 bettongs were lateralized. Analysis revealed that 11 subjects (92%) displayed left forelimb preference and one (8%) – right forelimb preference, thus, there was a significant prevalence of the left-forelimb preferent individuals (binomial test: *z* = 2.73; *p* = 0.006; Fig. 1c).

**Table 1:** Individual forelimb preferences in brush-tailed bettongs.

subject	sex	feeding on								tripedal stance				nest-material collecting		
		non-living food				live insects										
		HI	*z*	pref		HI	*z*	pref		HI	*z*	pref		HI	*z*	pref
1	M	0.81	4.49	L		0.80	5.06	L		0.73	5.18	L		0.79	4.27	L
2	M	0.55	3.05	L		0.55	3.48	L		0.33	2.38	L		0.72	3.90	L
3	M	0.42	2.33	L		0.40	2.53	L		−0.22	−1.54	N		−0.24	−1.30	N
4	M	0.68	3.77	L		0.70	4.43	L		0.65	4.62	L		0.86	4.64	L
5	M	0.42	2.33	L		0.50	3.16	L		0.14	0.98	N		0.59	3.16	L
6	M	0.48	2.69	L		−	−			0.22	1.54	N		−	−	L
7	M	0.74	4.13	L		−	−			0.49	3.50	L		0.45	2.41	L
8	F	0.68	3.77	L		0.65	4.11	L		0.53	3.78	L		0.72	3.90	L
9	F	0.87	4.85	L		0.70	4.43	L		0.69	4.90	L		0.86	4.64	L
10	F	−0.48	−2.69	R		−0.40	−2.53	R		−0.37	−2.66	R		−0.66	−3.53	R
11	F	0.74	4.13	L		0.65	4.11	L		0.61	4.34	L		0.79	4.27	L
12	F	0.61	3.41	L		0.45	2.85	L		0.02	0.14	N		0.52	2.79	L

HI: handedness index; *z*: *z* score, positive values indicate leftward bias, negative values indicate rightward bias; pref: forelimb preference, L: left forelimb; R: right forelimb; N: non-preferent.

Forelimb use during catching live insects was assessed for ten individuals from the Berlin Zoo, only (Table 1). We obtained 40 unimanual acts per individual in this type of behaviour. All 10 subjects were lateralized. The majority of individuals showed preference of the left forelimb (binomial test: *z* = 2.02; *p* = 0.022): nine bettongs (90%) showed preferences for the left forelimb and one (10%) was the right-forelimb preferent individual (Fig. 1c).

Unimanual forelimb-use during the nest-material collecting was assessed in 11 subjects (Table 1). We obtained 29 unimanual acts per individual in this type of behaviour. More subjects were lateralized, than displayed no significant forelimb preference (binomial test: *z* = 2.52; *p* = 0.012). Nine subjects (82%) were classified as left-forelimb preferent, one subject (9%) – as right-forelimb preferent, and one individual (9%) had no preference (Fig. 1c). This distribution differed significantly from chance (*χ* ^2^_2_ = 19.00, *p*<0.001); and among lateralized subjects significantly more bettongs displayed the left-forelimb preference (binomial test: *z* = 2.52; *p* = 0.012).

Table 1 shows the individual forelimb preferences for supporting the body in the tripedal stance. After reducing data on each subject to the least value in the group, 51 unimanual act per individual was obtained. The distribution of lateralized and non-lateralized individuals did not differ significantly from chance (binomial test: *z* = 0.86; *p* = 0.388). Seven out of 12 bettongs were left-forelimb preferent (58%), one – right-forelimb preferent (8%), and other four individuals showed no significant bias in the forelimb use (33%) (Fig. 1c). This distribution differed significantly from chance (*χ* ^2^_2_ = 7.33, *p* = 0.026).

### 2. Direction of Laterality

The direction of forelimb preferences in all studied types of behaviour was not influenced by subjects’ sex (Mann–Whitney *U* tests: feeding on non-living food: *U* = 14.00, *p* = 0.624; catching live insects: *U* = 10.50, *p* = 0.753; nest-material collecting: *U* = 14.50, *p* = 1.00; supporting of the tripedal stance: *U* = 17.00, *p* = 1.00). The type of behaviour also had no effect on the direction of manual laterality (Friedman’s test: χ^2^_3_ = 3.00, *p* = 0.392).

**Figure 2:**
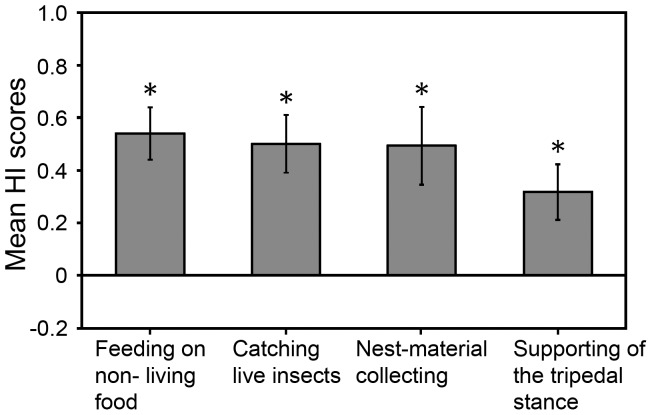
Direction of limb preferences for four types of unimanual behaviour investigated. Mean HI scores ±SEM for feeding on non-living food (*n* = 12), catching live insects (*n* = 10), nest-material collecting (*n* = 11) and supporting of tripedal stance (*n* = 12) (positive values indicate left lateral bias, negative values indicate right lateral bias). Significant group-level preference of the left forelimb was revealed in all types of behaviour. *p<0.05.

Significant group-level preference of the left forelimb was found for feeding on non-living food (mean HI±SEM = 0.54±0.10; one-sample Wilcoxon Signed-rank test: *Z* = 2.75, *p* = 0.006), for catching live insects (mean HI = 0.50±0.11; *Z* = 2.60, *p* = 0.009), for nest-material collecting (mean HI = 0.49±0.11; *Z* = 2.60, *p* = 0.009), and for supporting the body in the tripedal stance (mean HI = 0.32±0.11; *Z* = 2.76, *p* = 0.023) (Fig. 2).

### 3. Strength of Laterality

The analysis failed to reveal any effect of sex on the strength of manual preferences (Mann–Whitney *U* tests: feeding on non-living food: *U* = 11.50, *p* = 0.368; catching live insects: *U* = 11.00, *p* = 0.833; nest-material collecting: *U* = 11.50, *p* = 0.581; supporting of the tripedal stance: *U* = 15.00, *p* = 0.745). However, we found a significant influence of behavioural type on manual laterality (Friedman’s test: χ^2^_3_ = 9.12, *p* = 0.028), with stronger forelimb preferences for nest-material collecting than for supporting the body in the tripedal stance (post hoc Dunn’s test: *p*<0.05).

### 4. Consistency Across types of Behaviour

Significant correlation was found for bettongs’ forelimb preferences across all studied types of behaviour (feeding on non-living food vs. catching live insects: *r_s_* = 0.90, *n* = 10, *p*<0.001; feeding on non-living food vs. nest-material collecting: *r_s_* = 0.71, *n* = 11, *p* = 0.016; feeding on non-living food vs. supporting of the tripedal stance: *r_s_* = 0.75, *n* = 12, *p* = 0.005; catching live insects vs. nest-material collecting: *r_s_* = 0.94, *n* = 10, *p*<0.001; catching live insects vs. supporting of the tripedal stance: *r_s_* = 0.82, *n* = 10, *p* = 0.005; supporting of the tripedal stance vs. nest-material collecting: *r_s_* = 0.69, *n* = 11, *p* = 0.019).

## Discussion

The present study showed that brush-tailed bettongs displayed significant left-forelimb preference at the group level in four different types of their usual behaviour where one forelimb is used. The subjects exhibited strong individual biases, which were consistent between the tasks. In three types of unimanual actions (nest-material collecting, feeding on non-living food, and catching a live prey) significantly more bettongs were lateralized in the same direction: they preferred to use their left forelimbs. The direction of manual preferences was not affected by either animals’ sex or behavioural type. However, the type of task, though not the sex, influenced the strength of lateral biases. One may expect the division of labor between the forelimbs, that is in supporting themselves in the tripedal position bettongs would prefer to use the forelimb contralateral to the one preferred during manipulation. Such a specialized hand use has been previously assumed to be a characteristic of primates (e.g., [53], [54], [55]). In contrast, bettongs preferably use the same left forelimb for both the supportive and the manipulative tasks. Probably, functional specialization of forelimbs does not appear in bettongs, simply because they do not manifest manipulative abilities comparable with those seen in primates, and do not usually need the complementary involvement of two forelimbs in performing of a manual task. Furthermore, being a highly bipedal species [52], brush-tailed bettongs typically perform unimanual actions from the bipedal position. That is, the forelimb, which is not involved in manipulation, does not either provide the body support and remains passive still in the tripedal stance. The same direction of preferences in different types of actions may be associated with the lack of the need for manipulation with simultaneous postural support with a forelimb. Since positive correlation was found between the forelimb preference during manipulative tasks and the body support in the tripedal stance, we are discussing all the four types of behaviour in a single context.

In primates the bipedal posture was found to be attended with enhanced manual laterality at both within-subject and between-species levels [26], [30], [35]. The subjects performing the unimanual actions when staying bipedally demonstrate increased laterality as compared to the quadrupedal stance (e.g., [29], [31], [33], [34], [36], [38]) and, furthermore, the species with more bipedal and vertical postural habit tend to exhibit more pronounced individual- and population-level preferences than the quadrupeds (e.g., [26], [30], [35], [39], [40]). Recently, the hypothesis was proposed that in marsupials the body posture interacts with the expression of manual preferences in a similar way [21], [49]. Results of the present study in brush-tailed bettongs fully fit this line of evidence, i.e., the primary bipedal marsupial shows strong individual forelimb preferences and the unilateral directional bias at the group level. Previously it was found that the red-necked wallabies, which similarly to bettongs are characterized by habitual bipedalism, displayed population-level preferences in tasks initiated from the bipedal position [49]. In contrast, three obligatory quadrupedal marsupials: brush-tailed possums, grey short-tailed opossums, and sugar gliders showed individual, but not population biases in forelimb use [21], [50]. Since both studied marsupials with a bipedal locomotion, contrary to three quadrupeds, displayed group preferences, we suppose that bipedality can predict a group-level motor laterality in marsupials.

In quadrupedal grey short-tailed opossums and sugar gliders the direction of forelimb preferences was found to be strongly sex-related [21], whereas in brush-tailed bettongs as well as in red-necked wallabies we failed to reveal any significant sex effect on laterality. Probably, in marsupials strong sexual dimorphism in forelimb preferences, which hinder the expression of population bias, is a characteristic of quadrupeds, but not bipeds at least across studied species. Notably, among placentals the most contrasting sex-related differences in manual laterality have been reported also for obligatory quadrupeds such as horses [56], Mongolian gerbils [57], domestic cats [19], and dogs [58], [59].

**Figure 3:**
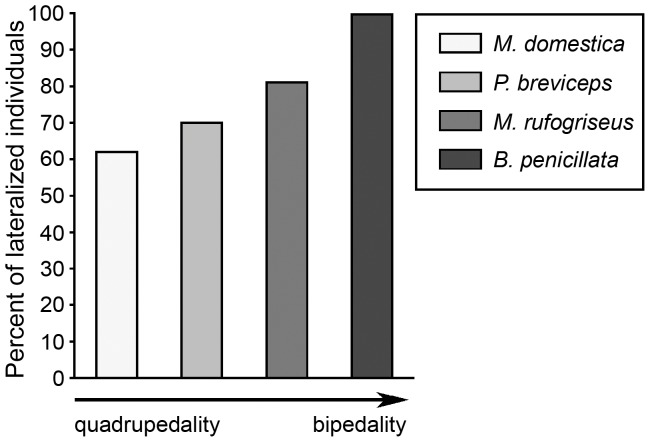
Percentage of lateralized individuals in marsupial species characterized by different degree of bipedality. The bars represent the percent of the subjects showed significant forelimb preferences for unimanual feeding on non-living food in grey short-tailed opossums, *Monodelphis domestica* (*n* = 26) [13], sugar gliders, *Petaurus breviceps* (*n* = 23) [13], red-necked wallabies, *Macropus rufogriseus* (*n* = 27) [49], and brush-tailed bettongs, *Bettongia penicillata* (*n* = 12) (the present study). The arrow indicates the increase of relative degree of bipedality and body verticality from grey short-tailed opossums – terrestrial quadrupeds rarely observed in a bipedal position and sugar gliders – arboreal quadrupeds frequently feeding in a bipedal position [13] to red-necked wallabies – bipedal saltators, but moving quadrupedally at slow speeds and brush-tailed bettongs, which prefer bipedal hopping even at the slow speed (see Discussion for a more detailed comparison).

**Figure 4:**
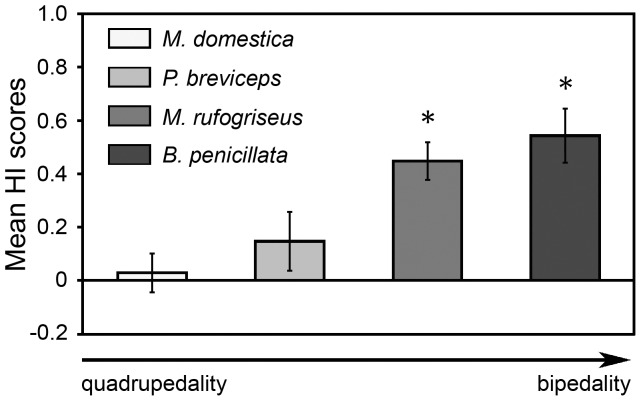
Comparative degrees of forelimb preferences at the group level in marsupial species characterized by different degree of bipedality. The bars represent mean HI scores ±SEM for unimanual feeding on non-living food in grey short-tailed opossums, *Monodelphis domestica* (*n* = 26) [13], sugar gliders, *Petaurus breviceps* (*n* = 23) [13], red-necked wallabies, *Macropus rufogriseus* (*n* = 27) [49], and brush-tailed bettongs, *Bettongia penicillata* (*n* = 12) (the present study). Asterisks indicate that the mean HI score differed significantly from zero, *p<0.05. The arrow indicates the increase of relative degree of bipedality and body verticality across marsupial species (see Fig.3 caption). The increase of bipedality across species corresponds with the increase of the degree of group-level preference.

According to McGrew and Marchant’s classification of manual preferences in primates [60] when the majority of subjects demonstrate the predominant use of one hand in most tasks this may be described as handedness. The studied sample of brush-tailed bettongs comprised a high percentage of individuals lateralized in the same direction, in particular, preferred to use their left paws (82–92%) in manipulation tasks (feeding on non-living food, catching insects, nest-material collecting). Such pronounced expression of lateralization, but at the population level, could be termed handedness. In primates the expression of pronounced manual laterality is usually associated with the bipedality [26], [30], [35], [38]. Brush-tailed bettongs use bipedal locomotion as a primary gait [52] and this is likely the most bipedal marsupial species studied to date in terms of forelimb preferences. In contrast to the other studied bipedal saltator, red-necked wallaby, bettongs were never observed manipulating food or nest material from initial quadrupedal position in captive conditions (our observations). Notably, at slow speeds macropodids such as wallabies normally move on all four legs, whereas bettongs usually use bipedal hopping even at slow speed [51], [52]. Thus, brush-tailed bettongs are characterized by a higher degree of bipedality than previously studied red-necked wallabies and apparently than the grey short-tailed opossums and sugar gliders, which both are quadrupeds. Meanwhile, bettongs have a higher percentage of lateralized individuals (100%) then wallabies (81%), gliders (70%), and opossums (62%) in feeding on non-living food (Fig. 3) – the most comparable type of unimanual behaviours between the studied species [21]. Furthermore, Figure 4 illustrates that the degree of forelimb preference at the group level is enhanced in species with more pronounced bipedality and more vertical body orientation. Together with previous studies our new findings support the hypothesis that in marsupials the degree of motor laterality expression correlates with the degree of bipedality in a species. Basing on this assumption, the most pronounced forelimb preferences could be expected in the entirely bipedal marsupial such as burrowing bettong, *Bettongia lesueur* [61], as well as in some extinct species, e.g., of the genera *Sthenurus* and *Procoptodon*, which were considered to be highly specialized bipedal hoppers and had very little or no dependence on quadrupedal locomotion [62].

It has been proposed that assumption of unstable bipedal posture requires increased activity and integration of the nervous system for balance control, and this arousal, in its turn, is reflected in the increase of motor laterality [28], [30]. Humans, however, are well-adapted obligate bipeds, which possessed balanced bipedal locomotion (e.g., [63], [64]), and meanwhile they do show the strong predominance of right-handedness across cultures and throughout history [23], [24], [22]). Like humans, marsupial bipedal hoppers have a number of specialized adaptations that allow them to adopt the stable bipedal stance; these are, for instance, notable disproportion between fore- and hindlimbs, as well as modifications in hindlimb skeleton such as elongation of the femur, tibia and metapodium [62], [65]. Thus, both humans and marsupial bipeds, such as red-necked wallabies and brush-tailed bettongs, can easily maintain the bipedal posture and are characterized by pronounced forelimb preferences. This demonstrates that even without the direct instability effect, the bipedality is attended by enhanced manual laterality in a species. One potential explanation for such a phenomenon is that the instability impacted on the laterality expression in the course of evolution of the very first bipeds, which might not yet have been morphologically adapted to bipedality. Alternatively, enhanced manual laterality in bipeds could be associated with a decreased involvement of forelimbs in locomotion. In most cases quadrupedal locomotion requires symmetrical effort of the left and right limbs that can potentially hinder the predominance of one forelimb over the other in unimanual actions. In marsupial bipedal hoppers, which have little dependence on quadrupedal locomotion, the forelimbs became free of constrains associated with locomotor activity that may be associated with the enhanced expression of pronounced laterality in forelimb use.

In red-necked wallabies the within-subjects postural effect on forelimb preferences have been previously found [49]. For example, when reaching for food was initiated in the bipedal stance wallabies showed strong individual- and population-level preferences, whereas in feeding from the quadrupedal posture no population bias was found and only few subjects showed individual preferences. Unlike wallabies, brush-tailed bettongs performed unimanual manipulations with food only from initial bipedal position; hence, it is difficult to trace the effect of posture here. However, prior to take the tripedal stance, bettongs stood on all four limbs and then raised one of the forelimb in the air. Thus, supporting of the tripedal stance can be classified as unimanual behaviour performed from the quadrupedal position. Notably, this was the only type of behaviour where the number of lateralized and non-lateralized bettongs did not differ significantly and the proportion of lateralized individuals (67%) was the lowest among all the behaviours studied. In addition, in supporting of the tripedal stance the strength of individuals’ lateral biases was significantly weaker than in nest-material collecting – the manipulative task performed by bettongs from the bipedal position. Altogether, these results may indicate that the quadrupedal stance slightly reduces the expression of forelimb preferences in brush-tailed bettongs. Nonetheless, in supporting of the tripedal stance significant group-level bias was still present and the distribution of individual preferences differed more than would be expected by chance. We suppose that in the extremely bipedal species, such as bettongs, motor laterality expresses even in the quadrupedal posture. Similarly, the population-level bias toward the use of the right hand in humans was showed for both quadrupedal and bipedal reaching [26]. The opposite tendency could also be traced: quadrupedal primates, gray mouse lemurs, showed no population hand preference for food grasping either from quadrupedal or bipedal body posture, and individual biases were not strengthened by unstable posture [40]. The same is true for non-primate obligate quadrupeds, such as domestic cats or tree shrews, in which unstable clinging or bipedal position also did not facilitate paw preferences [47], [48]. It could be assumed that in quadruped species, for which the bipedal stance is atypical, even evoked bipedality is not attended by the enhanced manifestation of forelimb preferences and vice versa, in bipeds the quadrupedal posture do not hinder the motor laterality.

To conclude, the present study shows that brush-tailed bettongs, the marsupials moving primarily by bipedal hopping, exhibit pronounced forelimb preferences at the individual and group levels. In contrast to quadrupedal marsupials, bettongs do not display sex differences in laterality. Our findings, together with previous reports, show that in marsupials bipedalism may predict the group-level directional bias for using a preferred hand in a species. Furthermore, interspecies comparison indicates the degree of motor laterality expression correlates with the degree of bipedality (see Fig.3, 4). All in all, our results provide evidence in support of the hypothesis that manual laterality expression in mammals is linked with species-typical postural habit.

## Materials and Methods

### 1. Subjects

Twelve brush-tailed bettongs, *Bettongia penicillata* were observed during the course of the study. Animals observed included 10 subjects (five males, #1–5 and five females, #8–12 in Table 1) from breeding colony of the Berlin Zoo, Germany and 2 subjects (males, #6, 7) from the Dortmund Zoo, Germany. All animals were captive born. Subjects from the Berlin Zoo lived in two mixed-sex groups of seven and three individuals in two different enclosures, while two males from the Dortmund Zoo lived together in one enclosure. To the best of our knowledge, close family relatedness was possible only within a few numbers of the subjects. The bettongs of Berlin Zoo were housed in inverted light/dark cycle (12h/12h), and animals from the Dortmund Zoo lived in normal light/dark cycle (12h/12h). Food (mainly consisted of chopped fruits, vegetables, nuts, and eggs) and fresh nest material (hay) were provided to animals daily at the beginning of the dark phase (Berlin Zoo) or at the end of the dark phase (Dortmund Zoo). Food and hay were placed randomly in a different location on each day of observation. In addition to the main diet the bettongs from the Berlin Zoo were fed with live insects (mealworms and locusts). The insects moved freely and actively scattered around the enclosure, therefore bettongs displayed prey-catching behaviour like in a natural foraging situation.

#### Ethics Statement

This study was conducted with pure observations on animals in two zoos, thus Declaration of Helsinki and Weatherall report are not applicable. At both zoos, the data collection did not lead to any changes in animals’ usual housing conditions and was provided with the permission of zoos’ administration. There was no physical contact between the observers and studied subjects.

### 2. Procedure

Data collection was conducted for 3–5 hours per day during 10 consecutive days in the Berlin Zoo and during four consecutive days in the Dortmund Zoo. We video recorded the unimanual behaviours of bettongs during the period of their normal activity (dark phase of light cycle) using two video cameras (Sony DCR-SR-220E and Sony DCR-HC-17E) in the NightShot mode with infrared lighting in black and white. In order not to affect the behaviour of animals, video recording was conducted from outside the enclosures. The video recording was carried out by two people simultaneously but of different subjects, which were active at the moment.

The video material was further analyzed frame-by-frame with Picture Motion Browser software Ver.3.0.00 (Sony Corp., Japan). Individual identification was based on natural markings (scars, individual features of colour etc.). We assessed preference in unimanual forelimb-use for four types of behaviour: feeding on non-living food (Fig. 1a), catching live insects, supporting of the tripedal stance (Fig. 1b), and nest-material collecting. To obtain discrete responses for each behavioral type after a single unimanual act was registered, the subsequent act was taken into account only if the individual moved to another location.

The unimanual feeding on non-living food, catching live insects, and nest-material collecting were performed by bettongs from initially bipedal position, i.e., standing on hind-limbs only with both forelimbs free. A unimanual act was counted when the subject reached for a non-living food item, an insect or nest material with one forelimb, leaving the other forelimb free. In contrast to other types of behaviour studied, unimanual supporting of the tripedal stance was performed from the quadrupedal position. Initially standing on all four limbs the subject raised one of the forelimbs, leaving the other one on the substrate. This posture resembles a tripedal stance previously described for marsupial quadrupeds [21] and gerbils [57]. The forelimb used by a subject for the body support in the tripedal stance was scored. Cases when the unimanual action was performed from a biased position (for instance, when the animal’s body was inclined to one side initially) were discarded from the analysis.

### 3. Data Analysis

In order to maximize comparability of forelimb-use scores across individuals, an equal number of unimanual acts per individual was obtained within each behaviour type before we applied the analysis [21], [49]. The minimal number of acts obtained per individual in the sample was estimated in the respective type of behaviour. Then, the first *n* acts equal to this number were taken from each individual data set. Thus, the number of unimanual forelimb-use acts in the respective type of behaviour was the same across individuals.

According to a Kolmogorov-Smirnov test, our data were not normally distributed. For this reason, we used nonparametric tests (two-tailed) for all analyses. Individual forelimb preference in each type of behaviour was determined by comparing the number of times a subject used the right or the left forelimb with a binomial test. As a result, the individuals were classified as having left-forelimb preference (positive *z* scores), right-forelimb preference (negative *z* scores), and having no preference of particular forelimb (*z* scores close to zero). To explore whether the distribution of left-preferent, right-preferent and non-preferent individuals differed significantly from chance a chi-square test was performed. A binomial test was used to evaluate the significance of difference between the number of lateralized and non-lateralized individuals, as well as the number of left-preferent and right-preferent individuals.

Further, an individual handedness index (HI) was calculated for each subject in each type of behaviour using the formula: (left forelimb use – right forelimb use)/(left forelimb use+right forelimb use). HI reveals the direction of preference and ranges from –1.0 to +1.0, with negative scores indicating the right forelimb bias and positive scores – the left forelimb bias. We used the absolute value of each subject’s HI (ABS–HI) to characterise the strength of individual forelimb preference independently of its direction.

The forelimb preference at the group-level was investigated with one-sample Wilcoxon Signed-rank test on individual HI scores. The influence of such factors as sex and type of unimanual behaviour was explored (on the basis of individual HI scores for direction and ABS–HI scores for strength) using Mann–Whitney *U* test and Friedman’s test with post hoc Dunn’s tests. Finally, we used the Spearman rank correlation to examine consistency of individual forelimb preferences across all four types of behaviour. An alpha value at 0.05 was adopted for all analyses.
